# Oral mucosa tissue gene expression profiling before, during, and after radiation therapy for tonsil squamous cell carcinoma

**DOI:** 10.1371/journal.pone.0190709

**Published:** 2018-01-16

**Authors:** Mette Marcussen, Mads Sønderkær, Julie Støve Bødker, Maria Andersen, Søren Nielsen, Charles Vesteghem, Ilse Christiansen, Olav Jonas Bergmann, Martin Bøgsted, Karen Dybkær, Mogens Vyberg, Hans Erik Johnsen

**Affiliations:** 1 Department of Clinical Medicine, Aalborg University, Aalborg, Denmark; 2 Clinical Cancer Research Center, Aalborg University Hospital, Aalborg, Denmark; 3 Department of Haematology, Aalborg University Hospital, Aalborg, Denmark; 4 Department of Oncology, Aalborg University Hospital, Aalborg, Denmark; 5 Institute of Pathology, Aalborg University Hospital, Aalborg, Denmark; 6 School of Dentistry, Faculty of Health Sciences, Aarhus University; Aarhus, Denmark; University of Sheffield, UNITED KINGDOM

## Abstract

**Background:**

Radiation-therapy (RT) induces mucositis, a clinically challenging condition with limited prophylactic interventions and no predictive tests. In this pilot study, we applied global gene-expression analysis on serial human oral mucosa tissue and blood cells from patients with tonsil squamous cell cancer (TSCC) to identify genes involved in mucositis pathogenesis.

**Methods and findings:**

Eight patients with TSCC each provided consecutive buccal biopsies and blood cells before, after 7 days of RT treatment, and 20 days following RT. We monitored clinical mucositis and performed gene-expression analysis on tissue samples. We obtained control tissue from nine healthy individuals. After RT, expression was upregulated in apoptosis inducer and inhibitor genes, *EDA2R* and *MDM2*, and in *POLH*, a DNA-repair polymerase. Expression was downregulated in six members of the histone cluster family, e.g., *HIST1H3B*. Gene expression related to proliferation and differentiation was altered, including *MKI67* (downregulated), which encodes the Ki-67-proliferation marker, and *KRT16* (upregulated), which encodes keratin16. These alterations were not associated with the clinical mucositis grade. However, the expression of *LY6G6C*, which encodes a surface immunoregulatory protein, was upregulated before treatment in three cases of clinical none/mild mucositis, but not in four cases of ulcerative mucositis.

**Conclusion:**

RT caused molecular changes related to apoptosis, DNA-damage, DNA-repair, and proliferation without a correlation to the severity of clinical mucositis. *LY6G6C* may be a potential protective biomarker for ulcerative mucositis. Based on these results, our study model of consecutive human biopsies will be useful in designing a prospective clinical validation trial to characterize molecular mucositis and identify predictive biomarkers.

## Introduction

Treatment-related toxicity remains a major concern in patients with head and neck cancers [1,2], including squamous cell cancer of the tonsil (TSCC). The incidence of TSCC is increasing, due to a shift towards younger patients with human papilloma virus (HPV)-positive cancers [3,4]. Consequently, more survivors must live with both short- and long-term cancer treatment side effects, including mucositis, hypo-salivation, tissue fibrosis, and hypo-vascular bone [5–8].

For curative intentions, radiation therapy (RT) is a key modality, with or without surgery, combined with concomitant chemotherapy. Squamous cell carcinomas require a relatively large amount of radiation (60 to 70 Gray [Gy]) [9]. Recently, outcomes have improved with the advent of radio-sensitizers and intensity-modulated RT. However, mucositis remains an acute, painful side effect [10]. Mucositis appears clinically at a total dose of approximately 35 Gy (after about 2 weeks), and it gradually worsens with each dose delivered [11]. The incidence of mucositis is 85% in patients with head and neck cancers that require RT; of these, 37% require hospitalization, and of these 51% require a feeding tube [5,6]. The lack of predictability of who are severely affected is a significant clinical challenge [12]. Palliative interventions may relieve the side effects, but no preventive medications are available that can reduce mucositis, and no markers are available for pretreatment identification of patients likely to be severely affected [13]. Previous studies have shown that RT causes DNA damage and oxidative stress, which subsequently lead to activation of p53-induced radiotoxic pathways, apoptosis, and cell-cycle arrest [14–16]. Furthermore, DNA repair and damage response via MDM2 suppression of p53, was also reported a consequence of RT in addition to radiation fibrosis, and endothelial damage [17–18]. However, no studies have described a gene expression analysis of human mucosa [19].

Here, we describe a disease- and treatment-specific global gene expression (GGE) pilot study. We examined consecutive mucosa biopsies and peripheral blood cells collected from patients with TSCC during RT treatment. This study aimed to generate phenotypic data to document the feasibility of a novel in vivo model of consecutive human biopsies during RT treatment that might provide new biological knowledge of the molecular pathogenesis of severe mucositis and facilitate the identification of potential predictive biomarkers.

## Materials and methods

### Patients

The Committee on Health Research Ethics of the Northern Denmark Region (N-20100022) approved the clinical protocol for this study. Informed written consent was obtained from all patients, in accordance with the Declaration of Helsinki. Patients were enrolled from September 1, 2010, to April 30, 2013. Inclusion criteria were age ≥18 years, cancer-treatment naïve, and without uncontrolled competitive disease.

**Fig 1:**
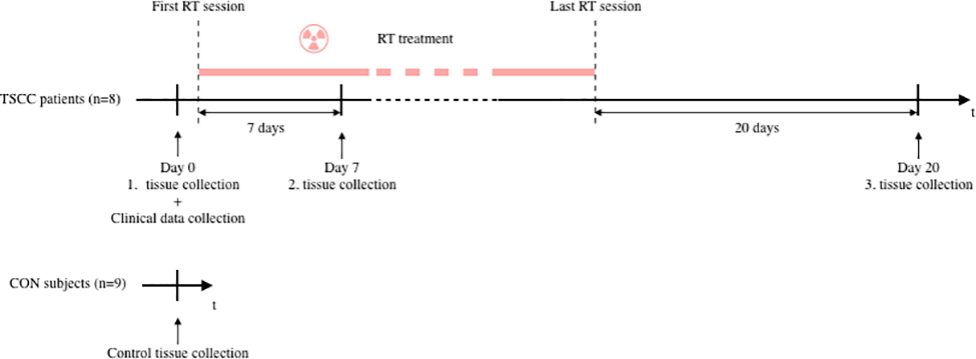
The pilot study design. Tissue samples were collected from patients with tonsil squamous cell cancer (TSCC) at three time points: at baseline, before RT (Day0), after 7 days of RT (Day7), and 20 days after the last RT. In addition, tissue samples were collected from healthy subjects (CON). All tissue collections consisted of one blood sample and one biopsy of oral buccal mucosa. Tissue samples were successively stored in our biobank. Once all the material was collected, gene expression analysis was performed collectively at the same laboratory.

We recruited 19 patients at the Department of Maxillofacial Surgery, Aalborg University Hospital. Among those patients, nine displayed histologically confirmed TSCC and a metastasis-negative FDG-PET/CT scan. Of these nine patients, seven provided three consecutive buccal biopsies and peripheral blood samples. The first biopsy and blood sample set (baseline) was acquired before the start of RT, the second set was acquired after one week of RT, and the third set was acquired at an average of 20 days after the last RT, for outline of the study plan (Fig 1). Two of the nine included patients died during treatment; one after the second biopsy and one before the first biopsy. The patient that died after the second biopsy was included in the analysis; thus, we analyzed eight patient samples. Our control group comprised 10 healthy, non-smoking individuals that had participated in a previous study [20]. Of these, one was excluded, due to an autoimmune disease that was not reported at the time of biopsy; thus, nine control samples were analyzed. All patients underwent pretreatment evaluations, including a medical history, smoking habits (smokers were defined as smoking more than 10 cigarettes per day), alcohol consumption (consumers were defined as drinking more than 21 units of alcohol weekly), and a clinical examination. Baseline characteristics were noted, including age, gender, and Eastern Cooperative Oncology Group (ECOG) performance status. Before RT, patients were screened for dental infections, and when indicated, infections were eradicated.

TSCC tumors were staged according to the TNM system for staging cancer (T = size of primary tumor; N = presence and level of lymph node involvement; M = presence of distant metastasis) [21]. Immunohistochemistry was performed to detect p16 overexpression in the tumor, which indicated HPV-induced TSCC. All patients with TSCC received intention-to-cure treatments. Accelerated external RT was applied in 6 weekly fractions of 2 Gy. RT was supplemented, when indicated, with concomitant cisplatin (40 mg/m^2^) once weekly during RT, according to international guidelines [22,23]. We noted the total radiation dose (Gy) applied to the tumor, based on the radiation schemes. We also calculated the estimated dose applied to the buccal mucosa at the site of the biopsy.

### Mucositis assessment

Oral mucositis (OM) was evaluated weekly in all patients, by the same researcher (MM), according to the World Health Organization oral toxicity assessment worksheet [24]. Subjective symptoms (pain and ability to eat solid food) and objective oral mucositis-related findings (erythema, ulceration) were noted. Grades 0 and 1 comprised none/mild mucositis (NM); this included soreness, with or without erythema, but solid food could be taken. Grades 2 to 4 comprised ulcerative mucositis (UM); in UM, food intake gradually declined, due to pain, and parenteral feeding might become necessary. The highest score measured during treatment was noted as the patients general mucositis experience.

### Collection of mucosa tissue and blood cells

Sample collection was performed with the methods described previously [20]. Briefly, a lens-formed, 5-mm biopsy was harvested with a scalpel from the buccal mucosa approximately 1 cm inferior to the parotid papilla in a standardized manner, and the wound was tightly sutured. One half of the biopsy was immediately embedded in RNA*later*™, for GGE analysis. The other half was fixed in formalin and embedded in paraffin for immunohistochemistry.

Within 2 h of the biopsy procedure, 15 ml of EDTA-mixed venous full blood was collected. Then, mononuclear cells were isolated and stored at -196°C in liquid nitrogen, until analysis.

### Gene expression analysis

Gene expression was evaluated with the methods described previously in detail [20]. Briefly, for both mononuclear cells and mucosa, we used the Affymetrix GeneChip Human Exon 1.0 ST Arrays with the Affymetrix GeneChip WT Terminal Labeling and Controls Kit (P/N 901524). CEL files were generated with Affymetrix GeneChip Command Console Software and deposited in the NCBI Gene Expression Omnibus repository, under number GSE103412.

### Immunohistochemistry

Tissue blocks were cut in 4-μm sections, and the sections were mounted on glass slides. With an in-house optimized protocol, tissues were stained for scinderin with a rabbit polyclonal antibody (KIAA1905, Nordic Biosite, www.nordicbiosite.com). Stained slides were scanned on a Hamamatsu NanoZoomer slide scanner and analyzed with NDP viewer software. To estimate the scinderin stain intensity, each stained slide was viewed at a magnification of ×15, and evaluated within a framed rectangle of 0.75 × 0.4 mm (0.3 mm^2^). Samples were classified as no stain (0), lightly stained (+), or heavily stained (++).

### Statistical analysis

All statistical analyses were performed with R [25] version 3.2.2 and Bioconductor packages [26].

#### Estimation of sample size

We applied the method described by Lee and Whitmore to identify genes that varied more than two-fold between test points, with a false discovery rate (FDR) less than 0.05% and a power of 90% [27]. This analysis was implemented in the R-package, size-power (Qui 2008). The results indicated that 10 patients in each group would provide sufficient statistical power.

#### Data preprocessing

The CEL files produced by the Affymetrix Expression Console were preprocessed and summarized at the gene level with the RMA algorithm in the Bioconductor package, affy, based on custom CDF-files [28]. This preprocessing step revealed the expression levels of 38,830 genes for each exon array. Genes were annotated with Ensembl gene identifiers.

#### Detection of differential expression

With the patient ID as a cluster variable, we used the limma package, a linear mixed model analysis provided in R, and the empirical Bayes approach to test for significant differences in gene expression between the second biopsy/blood sample and baseline, and between the third biopsy/blood sample and baseline [29]. To test for significantly differentially expressed genes between baseline and control samples, an unpaired t-test was performed with limma [29]. Patients were divided into two groups based on mucositis status (UM or NM), and significantly differentially expressed genes were detected with limma at each time point.

The FDR-adjusted P-values (≤0.05) were controlled with the Benjamini–Hochberg method [30] for each of the above tests.

#### Hierarchical clustering

The GGE data set of all nine control samples and the eight TSCC samples were divided into eight subsets. These subsets were gene biotypes (defined as protein coding), pseudogenes, miRNA, rRNA, snoRNA, snRNA, linRNA, and antisense transcript. Each dataset was subjected to hierarchical clustering, where the Pearson correlation was used as a distance measure, and average linkage was used as the algorithm method [31].

## Results

### Clinical characteristics of TSCC

The pilot study design is shown in Fig 1. Three steps of intervention were planned during TSCC-specific standard therapy, which included RT and cisplatin treatments. We collected 32 biopsies and performed 32 blood draws (7 × 3 sample sets + 1 × 2 sample sets for TSCC and 9 × 1 sample set for controls).

Patient clinical characteristics and demographics are shown in Table 1. Age was comparable between the control (age 58 years, range 47–78) and TSCC (age 63.5 years, range 51–69) groups. A trend towards male dominance was observed in the TSCC group (2 females among 8 patients), but not in the control group (4 females among 9 patients). Five of eight patients with TSCC were smokers, and four of the eight consumed alcohol. Tumor staging was evaluated according to the TMN system [21], and p16 overexpression was detected in six of eight tumors.

**Table 1:** Patient characteristics and demographics upon enrollment in the study.

Patient	Age	Gender	ECOG^a^	Smoking^b^	Alcohol^c^	Staging^d^	p16^e^
**TSCC01**	57	m	0	0	0	T1N2bM0	yes
**TSCC03**	67	f	2	1	1	T1N0M0	no
**TSCC04**	74	m	2	0	1	T1NxM0	no
**TSCC05**	72	m	0	0	0	T1N2aM0	yes
**TSCC06**	65	m	0	1	1	T1N1M0	yes
**TSCC07**	59	m	1	1	1	T2N1M0	yes
**TSCC08**	68	m	0	1	0	T4aN2cM0	yes
**TSCC09**	56	f	0	1	0	T2N2cM0	yes

^a^Eastern Cooperative Oncology Group (ECOG) performance status at baseline

^b^Smoking categories: 0 = Non-smoker, 1 = smoked more than 10 cigarettes per day

^c^Alcohol categories: 0 = No alcohol consumption, 1 = consumed more than 3 units of alcohol per day

^d^TNM system for staging of cancer: T = size of primary tumor; N = presence and level of lymph node involvement; M = presence of distant metastasis (1)

^e^Overexpression of p16 indicates positive for HPV

The clinical data collected during RT are shown in Table 2. After treatment initiation, the second biopsy was acquired at a median of 7 days (range 3–12), and the third biopsy was acquired at an average of 57 days (range 40–92). Two patients, TSCC06 and TSCC08, did not receive cisplatin. An average dose of 68.3 Gy (range 66–76) was applied to the tumors. According to the radiation schemes, a total dose of approximately 30–35 Gy was applied to the buccal mucosa bilaterally. At the time of the second biopsy, an average dose of 7.7 Gy (range 4.2–14.4) was applied. UM was detected in five patients and NM was detected in three patients. The median mucositis scores were 1.9 (range 0–3): 2.6 for the UM group and 0.7 for the NM group. We observed no signs of infection at the site of biopsy. All samples yielded valid gene expression profiles.

**Table 2:** Patient clinical data during radiation treatment and at follow-up.

Patient	Total dose of radiation to tumor	Estimated dose of RT at biopsy site day7	Cisplatin (40 mg/m^2^) once weekly during RT	WHO^a^	Days from treatment start to second biopsy	Days from second to third biopsy
**TSCC01**	66 Gy/33 fr	4.2	yes	3	3	53
**TSCC03**	66 Gy/33 fr	9.5	yes	0	8	42
**TSCC04**	68 Gy/34 fr	9.3	yes	1	8	dead
**TSCC05**	68 Gy/33 fr	14.4	yes	2	12	41
**TSCC06**	66 Gy/33 fr	5.3	no	1	4	40
**TSCC07**	68 Gy/34 fr	5.1	yes	3	4	52
**TSCC08**	76 Gy/56 fr	6.9	no	2	9	80
**TSCC09**	68 Gy/34 fr	7.2	yes	3	6	92

**Abbreviations:** Gy: Gray; fr: fractionated; WHO: World Health Organization

^a^mucositis stage, according to the WHO assessment scale, was measured weekly, during treatment and after, until mucositis dissolved [24].

### Gene expression in mucosa and blood

The differentially expressed genes in mucosa (Table 3) and blood (Table 4) are annotated with a gene symbol, the fold change (FC), the adjusted p-value, the gene ontology terms (GO-terms), and the gene function.

**Table 3:** Genes altered in mucosal tissue from patients with tonsil squamous cell carcinoma receiving radiation therapy.

Gene symbol	FC	p-value	adj. p-value	Qualified Gene Onotology term	Function
**Baseline**					
LIFR	-2.73	2.09e-05	0.019	Leukemia Inhibitory Factor Receptor Alpha	Cellular differentiation, proliferation, survival
FKBP5	-2.48	0.00015	0.037	FK506 Binding Protein 5	Immune regulation, basic cellular processes
SPARCL1	-2.24	0.0002	0.041	SPARC Like 1	Cell adhesion, migration, and proliferation
MS4A4E	-2.30	9.06e-06	0.018	Membrane Spanning 4-Domains A4E	Cell surface signaling
PDGFRA	-2.11	1.74e-06	0.010	Platelet Derived Growth Factor Receptor Alpha	Cell surface tyrosine kinase receptor
RN7SL783P	2.54	0.00010	0.031	pseudogene	Unknown function
MTND5P8	2.17	0.0002	0.04	pseudogene	Unknown function
ABO	2.02	8.82e-07	0.001	Alpha 1-3-N-Acetylgalactosaminyltransferase	Enzyme, modifying surface glycoproteins
**After seven days of radiotherapy**					
HIST1H3B	-2.91	7.52e-08	0.000143	Histone Cluster 1, H3b	Transcription
HIST1H2BM	-2.75	1.6e-07	0.000251	Histone Cluster 1, H2bm	Transcription
CYSLTR1	-2.54	3.91e-05	0.0098	Cysteinyl Leukotriene Receptor 1	Cell structure
HIST1H3C	-2.39	9.08e-06	0.0039	Histone Cluster 1, H3c	Transcription
HIST1H3H	-2.17	4.53e-08	0.000105	Histone Cluster 1, H3h	Transcription
MOXD1	-2.16	6.19e-08	0.000128	Monooxygenase DBH Like 1	Metabolism
HIST1H1A	-2.12	0.00016	0.022	Histone Cluster 1, H1a	Transcription
HIST1H1B	-2.09	1.05e-08	3.14e-05	Histone Cluster 1, H1b	Transcription
MKI67	-2.00	2.58e-06	0.0016	Marker Of Proliferation Ki-67	Transcription
WDR63	2.67	1.09e-10	1.1e-06	WD Repeat Domain 63	Unknown
MDM2	2.29	6.77e-11	4.26e-11	MDM2 oncogene, E3 ubiquitin protein ligase	Apoptosis
EDA2R	2.26	8.38e-11	1.0e-06	Ectodysplasin A2 receptor	Apoptosis
POLH	2.17	3.22e-10	1.81e-06	Polymerase; DNA directed	Transcription
KRT16	2.15	0.00058	0.052	Keratin 16	Cell structure
**Three weeks after RT cessation**					
ANKRD20A5P	-3.56	2.90e-07	0.0026	Ankyrin Repeat Domain 20 Family Member A5	Pseudogene
CYSLTR1	-3.11	3.92e-06	0.0082	Cysteinyl Leukotriene Receptor 1	Cell structure
SCIN	-2.50	9.09e-05	0.044	Scinderin	Cell structure
ANKRD20A11P	-2.47	4.93e-05	0.033	Ankyrin Repeat Domain 20 Family Member A11	Pseudogene
ANKRD20A9P	-2.32	1.2e-06	0.0052	Ankyrin Repeat Domain 20 Family Member A9	Pseudogene
CYP4F34P	-2.28	4.1e-05	0.032	Cytochrome P450 Family 4 Subfamily F Member 34	Pseudogene
TC2N	-2.13	6.47e-05	0.036	Tandem C2 Domains, Nuclear	Metabolism
IL1R2	-2.12	3.37e-07	0.0026	Interleukin 1 Receptor Type 2; cytokine receptor of the interleukin 1 receptor family	Immune response
MIR31HG	5.30	5.71e-05	0.035	Non-coding microRNA no 3	Non-coding mi-RNA
CCAT1	3.08	1.08e-05	0.018	Colon Cancer Associated Transcript 1	Non-coding RNA
PTPRZ1	2.93	0.000103	0.047	Protein Tyrosine Phosphatase, Receptor Type Z1	Transcription
**NM versus UM**					
LY6G6C	-3.78	2.53e-06	0.0995	Lymphocyte Antigen-6 G6C	Signal transduction Immune response

**Table 4:** Genes altered in mononuclear cells of the blood from patients with tonsil squamous cell carcinoma receiving radiation therapy.

Gene symbol	FC	p-value	adj. p-value	Qualified Gene Onotology term	Function
**Baseline**					
RNU6-620P	-11.8	1.48e-12	5.80e-08	RNA, U6 small nuclear 620, pseudogene	pseudogene
RNU6-422P	-3.77	3.03e-08	0.00022	RNA, U6 small nuclear 422, pseudogene	pseudogene
RNU6-737P	-3.36	1.34e-07	0.00034	RNA, U6 small nuclear 737, pseudogene	pseudogene
RNU6-795P	-2.85	2.82e-06	0.0024	RNA, U6 small nuclear 795, pseudogene	pseudogene
RPS7P2	-2.63	2.14e-07	0.00044	Ribosomal protein S7 pseudogene 2	pseudogene
AGAP9	-2.61	6.15e-06	0.0039	ArfGAP With GTPase Domain, Ankyrin Repeat And PH Domain 9	GTPase-activating
RNU6-336P	-2.45	5.72e-08	0.00025	RNA, U6 small nuclear 336, pseudogene	pseudogene
OAZ1	-2.26	6.81e-06	0.0040	Ornithine decarboxylase antienzyme 1	Cell growth and proliferation
RPL23AP64	-2.19	0.00012	0.018	Ribosomal protein L23a pseudogene 64	pseudogene
RNU6-1162P	-2.06	2.02e-05	0.0068	RNA, U6 small nuclear 1162, pseudogene	pseudogene
CCDC144B	-2.02	0.00074	0.043	Coiled-Coil Domain Containing 144B	pseudogene
RN7SL432P	-2.02	5.37e-07	0.00088	RNA, 7SL, cytoplasmic 432, pseudogene	pseudogene
RNU6-622P	7.30	7.74e-09	8.62e-05	RNA, U6 Small Nuclear 622, Pseudogene	pseudogene
DUTP6	3.45	1.74e-06	0.0019	Deoxyuridine Triphosphatase Pseudogene 6	pseudogene
SSU72P8	3.44	1.07e-07	0.0014	RNA Polymerase II CTD Phosphatase Homolog, Pseudogene 8	pseudogene
RNU6-919P	3.37	1.06e-05	0.0051	RNA, U6 Small Nuclear 919, Pseudogene	pseudogene
RPS6P15	3.01	2.82e-06	0.0024	Ribosomal Protein S6 Pseudogene 15	pseudogene
RN7SL748P	2.44	1.59e-05	0.0061	RNA, 7SL, Cytoplasmic 748, Pseudogene	pseudogene
RPL10P4	2.33	2.88e-07	0.00051	Ribosomal Protein L10 Pseudogene 4	pseudogene
RPL21P133	2.32	6.39e-07	0.0010	Ribosomal Protein L21 Pseudogene 133	pseudogene
RN7SL290P	2.22	1.06e-05	0.0051	RNA, 7SL, Cytoplasmic 290, Pseudogene	pseudogene
OR5M4P	2.21	4.97e-05	0.011	Olfactory Receptor Family 5 Subfamily M Member 4 Pseudogene	pseudogene
RNU6-151P	2.19	1.58e-07	0.00036	RNA, U6 Small Nuclear 151, Pseudogene	pseudogene
RNU6-135P	2.19	1.29e-07	0.00034	RNA, U6 Small Nuclear 135, Pseudogene	pseudogene
RNA5SP116	2.18	0.00085	0.046	RNA, 5S Ribosomal Pseudogene 116	pseudogene
NUTM2D	2.13	0.00016	0.021	NUT family member 2D	unknown
RNA5SP54	2.06	8.49e-08	0.00030	RNA, 5S Ribosomal Pseudogene 54	pseudogene
RN7SL865P	2.05	0.00074	0.043	RNA, 7SL, Cytoplasmic 865, Pseudogene	pseudogene
RPS29P8	2.00	8.69e-07	0.0012	Ribosomal Protein S29 Pseudogene 8	pseudogene

Before RT was applied, eight genes were altered in patients with TSCC compared to controls (Table 3). Five of these genes remained unaffected with subsequent therapy: *LIFR*, *FKBP5*, *SPARCL1*, *MS4A4E*, and *PDGFRA*.

In response to treatment, we found nine downregulated genes. Eight of these genes were in the histone cluster family, including *HIST1H3B*, *HIST1H2BM*, *HIST1H3C*, *HIST1H3H*, *HIST1H1A*, and *HIST1H1B*; and one, *MKI67*, was a marker of Ki-67 proliferation. Five genes were upregulated. Of these, two were related to apoptosis, *MDM2* and *EDA2R*, and one, *POLH*, encoded a transcriptional DNA-directed polymerase (Table 3).

**Fig 2:**
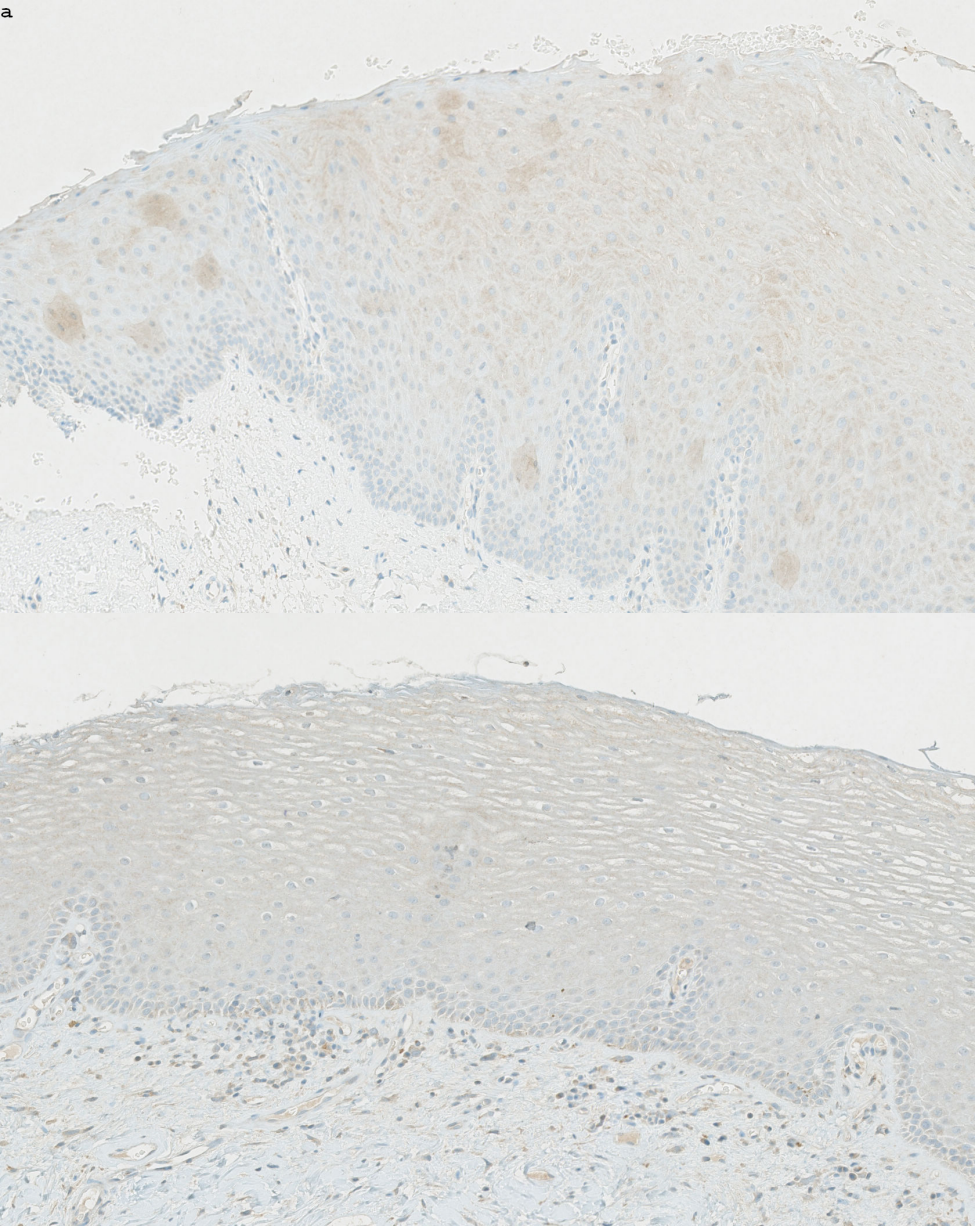
Immunohistochemical analysis of mucosal tissue expression of scinderin. Oral buccal mucosa section (×15 magnification) stained with an *SCIN* antibody. (a) High scinderin expression is evident in mucosa from a control individual (patient CON05). (b) Low scinderin expression is evident in mucosa from a patient with tonsil squamous cell cancer (patient TSCC07); the biopsy was acquired Day20. *SCIN* encodes a regulatory protein involved in exocytosis and we expected to se downregulation in salivary gland tissue, however epithelial cells were heavily stained in the healthy control group.

On day 21 after the last RT application, we found 11 altered genes compared to baseline (Table 3). Most were pseudogenes, including *ANKRD20A5P*, *ANKRD20A11P*, *ANKRD20A9P*, and *CYP4F34P* (downregulated); and noncoding RNAs, *CCAT1* and *MIR31HG*. The *MIR31HG* RNA was upregulated only among patients with TSCC that received cisplatin. *IL1R2* (downregulated) encoded a cytokine receptor of the interleukin 1 receptor family. *SCIN* (downregulated) encoded a regulatory protein involved in exocytosis. Immunohistochemical analysis results (Fig 2) showed reduced expression in epithelial cells, but not in the salivary glands.

**Fig 3:**
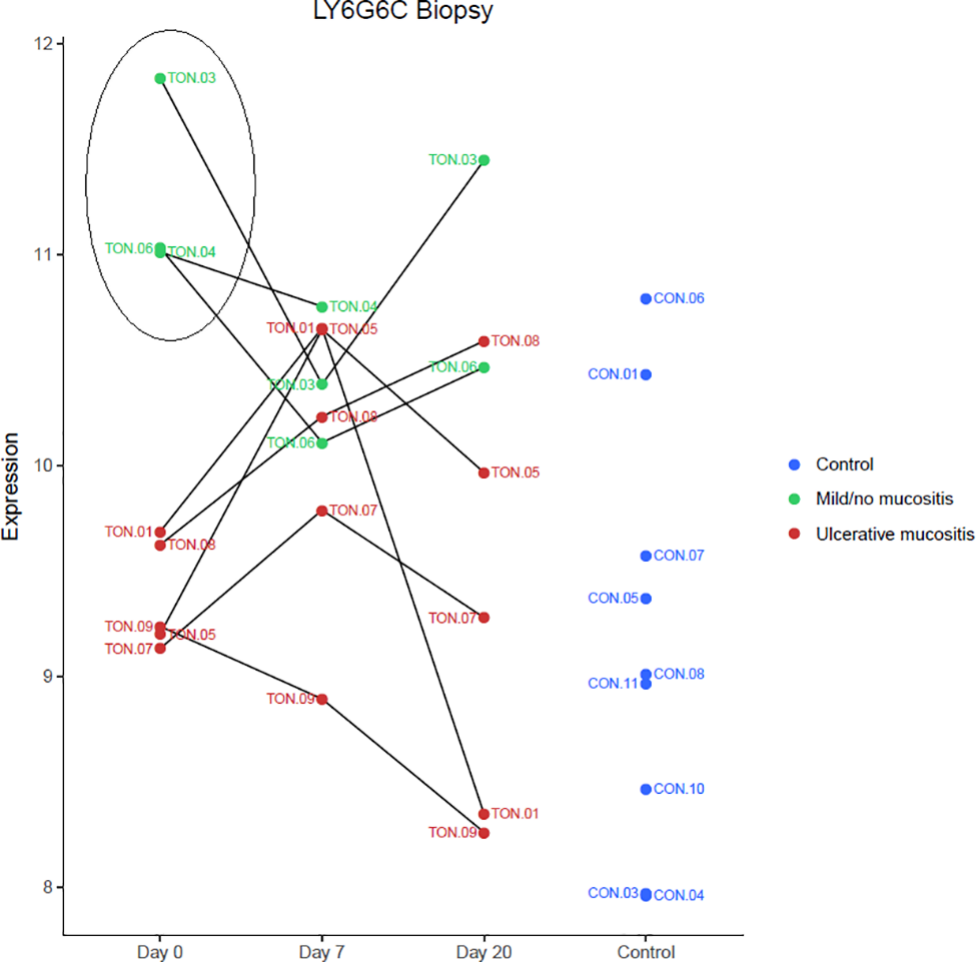
Expression of *LY6G6C* in mucosa. Dot plot shows expression of *LY6G6C*, at baseline (Day 0), after 7 days of RT (Day 7), and 20 days after the last RT session (Day 20), among patients that developed ulcerative mucositis (red) or mild/no mucositis (green), and in controls (blue). Patients with mild/ no mucositis exhibited an upregulation of *LY6G6C*. *LY6G6C* encodes a surface immunoregulatory protein expressed on mucosal dendritic cells.

When gene expression profiles of the buccal mucosa were compared between UM and NM samples, we found no differentially expressed genes (adjusted P < 0.05) in either blood or mucosa. However, one gene, *LY6G6C*, tended to be expressed at low levels (FC -3.78; adj. P = 0.0995) in UM baseline biopsies **(**Fig 3, Table 3).

**Fig 4:**
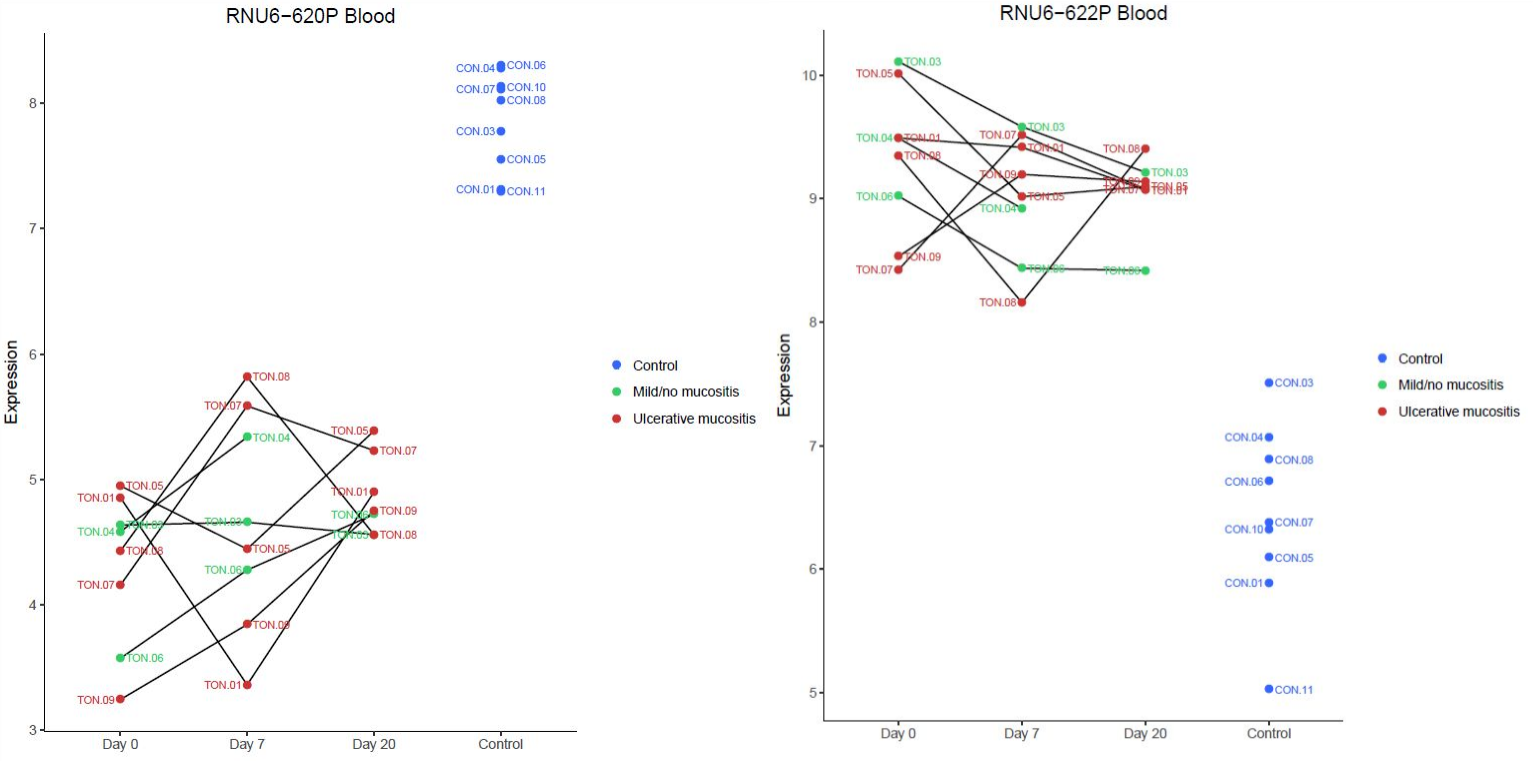
Expression of *RNU6-620P* and *RNU6-622P* in blood cells. Dot plot shows expression of *RNU6-620P* (FC-x11.8; P = 5.80e-80) and *RNU6-622P* (FCx7.3 P = 8.62e-05) at baseline (Day 0), after 7 days of RT (Day 7), and 20 days after the last RT session (Day 20), among patients that developed ulcerative mucositis (red) or mild/no mucositis (green). Expression in normal controls is indicated with blue circles. These genes encode small nuclear RNAs, which are non-protein coding genes. Patients with TSCC expressed a significant different level of both genes compared to healthy controls.

We found 12 downregulated and 17 upregulated genes in blood samples from the TSCC group compared to the control group (Table 4). These genes were dominated by small nuclear RNAs (snRNAs), e.g., *RNU6-620P* and *RNU6-622P*, and a dot plot of these two selected genes is shown in Fig 4.

**Fig 5:**
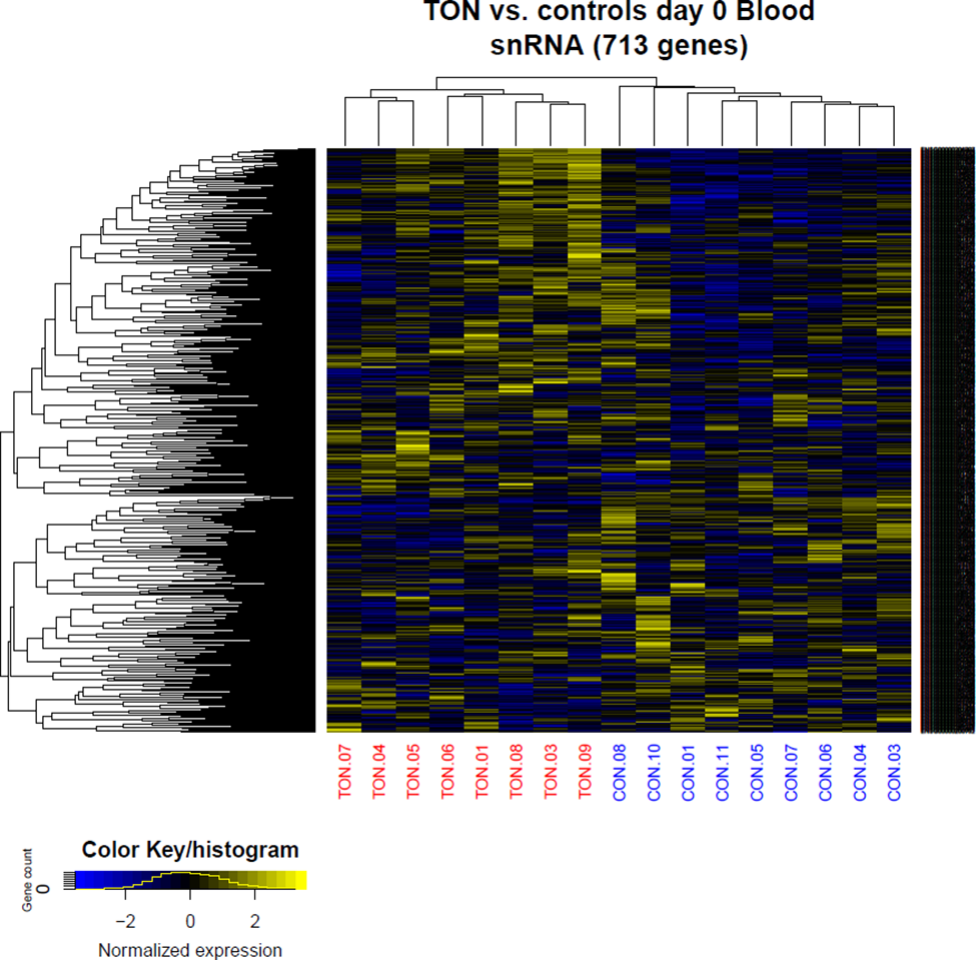
Small nuclear RNA (snRNA) clustered according to P value. The expression of snRNA regardless of fold change was clustered according to P value, showing a distinct division between patients with TSCC and healthy controls.

We clustered the expression levels of snRNAs, regardless of fold-changes according to P-value, and observed a distinct division between patients with TSCC and healthy controls (Fig 5). We found no differentially expressed genes in blood samples between baseline and day 7 or day 20.

## Discussion

This study aimed to validate our clinical pilot study set-up and demonstrate its potential for identifying pathogenic variables or biomarkers for molecular mucositis. In response to RT, we identified several altered genes in the mucosa, but no differentially expressed genes in the blood cells. Furthermore, the identified genes were not correlated to the grade of clinical mucositis. Furthermore, we found that although all patients with TSCC were diagnosed with a localized solid epithelial tumor, and the biopsies from the study group was harvested from clinically healthy buccal mucosa, the mucosal tissue and blood cells had different gene profiles compared to healthy controls before RT.

### RT effects on mucosal gene expression

Several studies have described the molecular effects of radiation on normal tissue, but no studies have described effects on gene expression levels [19]. Generally, short-term alterations include increased levels of p53 and other apoptotic markers (e.g., Bcl-2 and Mcl-1) [14,32], increased numbers of inflammatory cells (CD68-positive macrophages and other leukocyte subtypes), and alterations in inflammatory markers (e.g., NF-kB and COX-2) [32–35]. The epithelium begins to regenerate after one week of radiation, confirmed by the identification of cell proliferation markers, Ki-67 and [^3^H]-TdR, and by the increased expression of keratins (keratins 1, 6, 10, 16) [36,37]. Over the long term, RT caused different distribution patterns of adhesion molecules and macrophage subpopulations, compared to pretreatment specimens [38].

The present pilot study identified markers of apoptotic activity. *EDA2R* was upregulated in the mucosa 7 days after RT initiation, compared to its pretreatment status. *EDA2R* encodes the ectodysplasin A2 receptor, a transmembrane protein in the tumor necrosis factor receptor superfamily. Upon stimulation, this receptor activates the NF-ĸB and JNK apoptotic pathways [39]. In addition, six members of the histone cluster family were downregulated, which indicated DNA damage. Histones are basic nuclear proteins responsible for nucleosome structure. Previous studies in cell lines have described histone downregulation in response to RT [40].

In parallel, the *MDM2* oncogene (*MDM2*) was upregulated. *MDM2* encodes an E3 ubiquitin ligase, localized in the nucleus, and is regulated transcriptionally by p53. In turn, E3 ubiquitin ligase mediates the ubiquitination of p53, and thereby, inhibits p53-mediated cell-cycle arrest and apoptosis [41]. In addition, the upregulation of *POLH*, a polymerase that replicates UV-damaged DNA, indicated a DNA defense mechanism. Thus, we identified both inducers and inhibitors of apoptosis and DNA damage, consistent with findings reported in previous preclinical studies.

Proliferation-related genes were also altered. *MKI67*, which encodes Ki-67, a nuclear protein that is essential for cellular proliferation, was downregulated after 7 days of RT. This finding contrasted with findings from a previous study on human mucosa [32]. However, *KRT16* was upregulated. *KRT16* encodes keratin16, a protein characteristic of early differentiated epithelial cells. This short-term change indicated continuous epithelial proliferation [42]. This finding was also reported in a previous study [37].

*SCIN* encodes a calcium ion- and actin filament-binding protein with a regulatory function in exocytosis [43]. We expected *SCIN* to be associated with salivary gland function because of the connection to exocytosis function and prior studies have reported histological changes in radiated salivary glands, including atrophy, edema, cell death, and fibrous tissue formation [44]. However an immune histochemical stain for SCIN revealed that the presence was seen in the epithelial cells (Fig 2).

### A potential biomarker for the grade of clinical mucositis

When we compared samples from three patients with NM to samples from 4 patients with UM, we found that *LY6G6C* (lymphocyte antigen 6 complex, locus G6C) was upregulated (x3.78; P = 0.0995) in NM before treatment, although this finding was not statistically significant (Fig 3). *LY6G6C* belongs to a cluster of leukocyte antigen-6 genes linked to the major histocompatibility complex–class II. This complex is located at the cell surface, where it is involved in immune-mediated signal transduction.

In a previous study, we showed that two members of the major histocompatibility complex–class II gene family, *HLADR-B1* and *B5*, could potentially predict UM in patients with multiple myeloma [20]. We therefore hypothesize that HLA-based immunity protect against tissue inflammation during treatment in both these disease categories. Because mucositis may be considered an inflammatory state, those findings might add to our molecular understanding of why RT induces severe mucositis in some patients.

### Pretreatment gene signature of TSCC

In mucosa, we found that TSCC induced a specific gene signature different from controls (Table 3). In particular, we found that TSCC induced changes in the expression of leukemia inhibitor factor receptor-alpha (*LIFR*), platelet-derived growth factor receptor-alpha (*PDGFR*), and secreted protein acidic and cysteine rich-like protein (*SPARCL*) genes. First, this signature was present in clinically normal-appearing oral mucosa located at a distance from the tonsil tumor. Second, the signature was expressed independently of alcohol consumption, smoking habits, and p16 overexpression in the tumor, in addition to other clinical features. Third, this signature remained practically unchanged throughout RT.

*LIFR*, a transmembrane receptor protein of the type 1 cytokine receptor family, is involved in cellular differentiation, proliferation, and survival; moreover, it inhibits the p53 apoptotic pathway. Low expression has been detected in various human cancers [45]. However, *LIFR* has been identified as both a suppressor and a promoter of carcinogenesis. *PDGFR* encodes a cell-surface tyrosine kinase receptor that binds platelet-derived growth factor family members. The receptor complex activates pathways involved in cell migration and chemotaxis during wound healing [46]; additionally, mutations in *PDGFRA* play an active role in cancer development [47]. Finally, *SPARCL* is involved in extracellular matrix synthesis. It was downregulated in number human cancer types [48]. It remains unclear why these genes, which are involved in cellular differentiation, wound healing, and extracellular matrix formation, are downregulated in clinically normal-appearing mucosa acquired from patients with TSCC. Future studies should investigate whether this phenotype might indicate increased susceptibility to malignant transformation.

The GGE analysis of blood samples revealed a large array of snRNA-type pseudogenes. Of these, *RNU6-620P* was downregulated 11.8-fold (P = 5.80e-80) and *RNU6-622P* was upregulated 7.3-fold (P = 8.62e-05) compared to controls. A cluster analysis of the expression of snRNAs and other noncoding RNAs in the blood revealed that distinctly different clusters of noncoding RNAs were associated with TSCC and controls (Fig 5). The protein coding genes did not show the same distinction.

## Conclusion

In this pilot study, we described a gene signature expressed by mucosal tissue and circulating peripheral blood cells from patients with TSCC in response to RT. We chose to take the second biopsy before any macroscopic damage in order to gain insight into the molecular processes that underlie mucositis and to avoid harvesting disintegrates tissue dominated by inflammatory mediators. RT caused molecular alterations related to apoptosis, DNA damage, DNA repair, and proliferation. However, these alterations were independent of clinical mucositis severity. Furthermore we identified a potential protective biomarker for ulcerative mucositis. Based on these results, we concluded that our model was feasible, and the data will be useful in designing a prospective clinical validation trial for characterizing mucositis at the molecular level and identifying predictive biomarkers.

## Accesion codes

Phenotype data described in this manuscript was deposited at the NCBI Gene Expression Omnibus (GEO) repository under no GSE103412.
